# Lungenmanifestationen bei adulten rheumatischen Erkrankungen

**DOI:** 10.1007/s00393-020-00946-5

**Published:** 2021-01-04

**Authors:** S. Ewig, M. Bollow

**Affiliations:** 1 Klinik für Pneumologie und Infektiologie, Augusta-Kranken-Anstalt Bochum, Bochum, Deutschland; 2 Klinik für diagnostische und interventionelle Radiologie und Nuklearmedizin, Augusta-Kranken-Anstalt Bochum, Bergstr. 26, 44791 Bochum, Deutschland; 3 Ruhr-Universtät Bochum, Bochum, Deutschland

**Keywords:** Rheumatologische Erkrankungen, Lungenbeteiligung, HR-CT der Lunge, HR-CT-Muster, Interstitielle Lungenerkrankungen, Rheumatic diseases, Pulmonary involvement, HR-CT of the lungs, HR-CT patterns, Interstitial lung diseases

## Abstract

**Hintergrund:**

Rheumatologische Erkrankungen gehen häufig mit einer Lungenbeteiligung einher. Alle anatomischen Strukturen der Lunge können dabei betroffen sein. Für Lungengerüsterkrankungen gilt ein System der Muster, mit denen sie sich in der HR-CT der Lunge präsentieren. Rheumatologische Erkrankungen können unterschiedliche HR-CT-Muster aufweisen.

**Ziel der Arbeit:**

Systematische Beschreibung der Lungenbefallsmuster rheumatologischer Erkrankungen.

**Material und Methoden:**

Narratives Review auf dem Boden der aktuellen Literatur zum Thema aus rheumatologischer, pneumologischer und radiologischer Sicht.

**Ergebnisse:**

Eine Lungenbeteiligung ist häufig und prognostisch relevant. Die Zusammenfassung der Lungenbefallsmuster zeigt bei entzündlich rheumatologischen Erkrankungen eine hohe Variabilität hinsichtlich der bevorzugt befallenen anatomischen Strukturen als auch der bevorzugten Muster interstitieller Manifestationen. Prognostische Implikationen und wesentliche diagnostische Befunde werden synoptisch dargestellt.

**Diskussion:**

Jede hier aufgeführte rheumatologische Erkrankung kann mit einer Lungenbeteiligung einhergehen. Eine systematische diagnostische Evaluation ist daher bei Erstdiagnose und im Verlauf immer angezeigt. Neben Klinik und Lungenfunktion ist die HR-CT der Lunge für die Diagnostik entscheidend.

## Abstract (en)

**Background:**

Rheumatic diseases frequently present with pulmonary involvement. All anatomic structures of the lungs can be affected. Interstitial lung diseases are characterized by a system of patterns evident in high-resolution computed tomography (HR-CT) scanning of the lungs. The HR-CT pattern can differ between rheumatic diseases.

**Objective:**

Systematic description of all variants and patterns of pulmonary involvement in rheumatic diseases.

**Material and methods:**

Narrative review based on the current literature on the topic from the perspective of rheumatology, pulmonary diseases and radiology.

**Results:**

Pulmonary involvement is frequent and prognostically relevant. The summary of pulmonary involvement reveals a high variability of affected anatomical structures as well as patterns of interstitial diseases for inflammatory rheumatic diseases. A synopsis of the main diagnostic findings is provided.

**Conclusion:**

Every rheumatic disease presented here can be associated with pulmonary involvement. Therefore, a systematic diagnostic evaluation is mandatory at the first diagnosis as well as during follow-up. Apart from clinical findings and lung function HR-CT of the lungs is decisive for the diagnostics.

Die Häufigkeit der Lungenbeteiligung, v. a. der interstitiellen Lungenerkrankung bei rheumatologischen Erkrankungen macht eine Vertrautheit mit den verschiedenen Befallsmustern sowie der Systematik der HR-CT-Muster erforderlich. Es erfolgt eine systematische Zusammenfassung der Befallsmuster, ihrer Häufigkeiten, HR-CT-Muster und Prognose.

## Allgemeines

### Spektrum der Lungenmanifestationen rheumatischer Erkrankungen

Die Lunge als ein mit reichlich Bindegewebe ausgestattetes und stark durchblutetes Organ ist im Rahmen von rheumatischen Erkrankungen häufig und in allen ihren anatomischen Strukturen (Atemwege, Alveolen, Gefäßbett, Pleura) betroffen [1, 2]. Darüber hinaus kann auch die Atempumpe bzw. das Zwerchfell Teil der rheumatischen Erkrankung sein.

Jede rheumatische Erkrankung weist dabei ein spezifisches Spektrum an möglichen Befallsmustern auf, die sich zum Teil wiederum deutlich überschneiden. Die klinische Herausforderung besteht daher darin, eine adäquate Zuordnung der pulmonalen Befallsmuster zu treffen und in ihrer therapeutischen Konsequenz [10] zu bewerten. Dies kann nur interdisziplinär durch Radiologen, Rheumatologen und Pneumologen erreicht werden. Die Tab. 1 und 2 geben einen summarischen Überblick über die pulmonalen Befallsmuster rheumatischer Erkrankungen.

**Table 1:** 

Befallsmuster	RA	SLE	PSS	PM/DM/Jo‑1	SS	MCTD	AS
Atemwegsbefall	++	+	–	–	+	–	–
ILD	+++	+	+++	+	+	+	–
Noduli	+++	–	–	–	–	–	–
Apikaler zystisch-fibröser Umbau	+	–	–	–	–	–	++
Vaskulitis	+	++	+	–	–	+	–
Pulmonale Hypertonie	+	++	+++	+	+	+	–
Pleura	++	++	+	–	–	–	–
Befall der Atemmuskel- bzw. Zwerchfellmuskulatur	–	+	–	+++	–	+	–
Aspirationspneumonie	–	–	++	+	–	+	–

*RA* rheumatoide Arthritis, *SLE* systemischer Lupus erythematodes, *PSS* progressive systemische Sklerodermie, *SS* Sjögren-Syndrom, *MCTD* „mixed connective tissue disease“, *AS* ankylosierende Spondylitis, *ILD* interstitielle Lungenerkrankung

+ bestehende Assoziation, ++ häufige Assoziation, +++ häufige und typische Assoziation

**Table 2:** 

ILD-Muster	RA	SLE	PSS	PM/DM/Jo‑1	SS	MCTD	AS
DAD	–	+	–	+	–	+	–
DAH und Vaskulitis	+	++	+	+	–	+	–
OP	++	+	+	+	+	–	–
UIP	+++	+	+	+	–	+	+
NSIP	++	++	+++	++	–	+	–
Lymphoide Proliferationen (LIP)	+	–	–	–	+	–	–

*RA* rheumatoide Arthritis, *SLE* systemischer Lupus erythematodes, *PSS* progressive systemische Sklerodermie, *SS* Sjögren-Syndrom, *MCTD* „mixed connective tissue disease“, *AS* ankylosierende Spondylitis, *DAD* „diffuse alveolar damage“, *DAH* „diffuse alveolar hemorrhage“, *OP* „organizing pneumonia“, *NSIP* „nonspecific interstitial pneumonia“

+ bestehende Assoziation, ++ häufige Assoziation, +++ häufige und typische Assoziation

Über diese typischen Assoziationen hinaus muss klinisch bei allen rheumatologischen Erkrankungen immer mit seltenen Lungenmanifestationen gerechnet werden.

Die Differenzialdiagnose wird zusätzlich erschwert durch eine bestehende immunsuppressive Therapie, sei es in Form von infektiösen Pneumonien oder toxischen Lungenschäden. Zudem prädisponieren einige rheumatische Erkrankungen zu Aspirationspneumonien bzw. infektiösen Pneumonien im Rahmen einer gestörten Atemmuskel- bzw. Zwerchfellfunktion.

### Klassifikation der interstitiellen Lungenerkrankungen (ILD)

Die Klassifikation der ILD erfolgt mehrdimensional. Die ILD werden dabei eingeteilt in pathologisch-anatomische und CT-morphologische Muster sowie in klinische Gruppen.

Die pathologisch-anatomischen Muster wurden dabei in Konsensuskonferenzen festgelegt [12, 13].

Für die CT-Morphologie hat sich die Klassifikation nach Mustern durchgesetzt. Diese sind auch bei rheumatischen Erkrankungen von grundlegender Bedeutung und in Tab. 3 zusammengefasst. Gleiche Muster sind allerdings nicht gleichbedeutend mit identischen klinischen Verläufen und prognostischen Implikationen.

**Table 3:** 

CT-Kriterium	CT-Muster						
	UIP	Possible UIP^a^	NSIP	RB-ILD/DIP	LIP	AIP	OP
Verteilung basal betont	+	+	+	–	–	–	–
Subpleurale Aussparung	–	–	+	–	–	–	–
Retikulationen^b^	+	+	+	–	–	–	–
Traktionsbronchiektasen	+	+	+	–	–	–	–
Honigwaben	+	–	–	–	–	–	–
Milchglastrübungen^c^	–	–	+	+	+	+	+
Konsolidierungen	–	–	–	–	–	+	+
Zysten	–	–	–	–	–	–	–
Mosaikmuster	–	–	–	–	–	–	–

*UIP* „usual interstitial pneumonia“, *NSIP* „nonspecific interstitial pneumonia“, *RBILD* „respiratoy bronchiolitis-interstitial lung disease“, *LIP* „lymphoid interstitial pneumonia“, *AIP* „acute interstitial pneumonia“, *OP* „organizing pneumonia“

^a^Entscheidend in der Abgrenzung sind das Fehlen von Honigwaben sowie das Fehlen von Milchglastrübungen, Konsolidierungen, Zysten und eines Mosaikmusters

^b^Retikulationen werden als Kriterium bewertet, wenn diese gegenüber Milchglastrübungen überwiegen

^c^Milchglastrübungen werden als Kriterium bewertet, wenn diese gegenüber Retikulationen überwiegen

**Figure 1:**
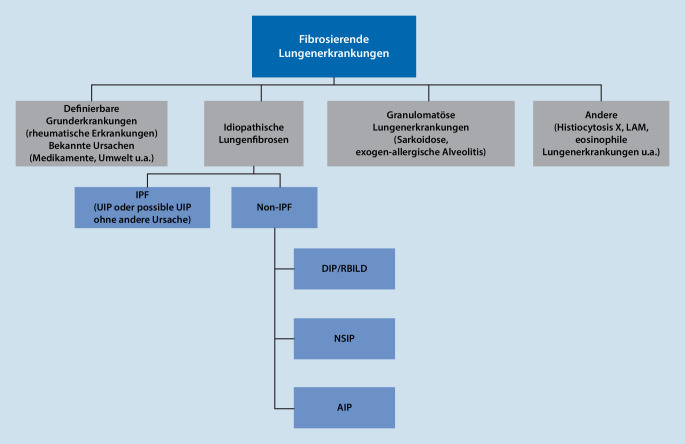

Klinisch ergibt sich eine Einteilung in 4 große Gruppen. Die Lungenmanifestationen der rheumatischen Erkrankungen [8] fallen in die erste Gruppe mit einer definierbaren Grunderkrankung oder bekannten Ursachen (Abb. 1).

### Interstitielle Lungenerkrankungen und rheumatische Erkrankungen: Formen der Assoziation

Die Rede von einer „Lungenbeteiligung bei rheumatischer Erkrankung“ könnte sich nach letzten Erkenntnissen in pathogenetischer Hinsicht als zumindest irreführend einseitig erweisen; so mehren sich die Hinweise, dass die Lunge auch Ausgangspunkt einer rheumatischen Erkrankung wie der RA oder der PSS sein kann [15, 2, 3, 4]. Manche Autoren sprechen bei solchen Verläufen auch von einer „lung dominant-connective tissue disease“ (CTD) [11].

Häufig wird bei rheumatischen Erkrankungen auch ohne manifeste ILD in der bronchoalveolären Lavageflüssigkeit eine lymphozytäre Alveolitis gefunden. Die Bedeutung dieses Befundes ist aktuell unklar. Patienten mit manifester ILD weisen meist zusätzlich eine Neutrophilie auf.

Umgekehrt weist eine Gruppe von Patienten (v. a. jüngere Frauen) mit manifester ILD (v. a. NSIP und OP) autoimmune Marker ohne manifeste rheumatische Erkrankung auf. Diese Gruppe wurde jüngst zu Forschungszwecken als „interstitial pneumonia with autoimmune features“ (IPAF) näher definiert [4].

Schließlich muss noch auf die Gruppe der subklinischen ILD hingewiesen werden (Diagnose über subtile Veränderungen in der CT), für die weder geklärt ist, ob sie ein Initialstadium einer manifesten ILD ist, noch, ob sie möglicherweise sogar ein Initialstadium einer rheumatischen Erkrankung darstellt.

Die Tab. 4 fasst diese komplexen möglichen Beziehungen zwischen rheumatischen Erkrankungen und Lungenmanifestationen zusammen.

**Table 4:** 

Subklinische ILD	Unklare Bedeutung
Manifeste ILD mit rheumatischer Erkrankung im Verlauf	Vor allem RA und PSS
Klassischer „Lungenbefall“ bei bekannter rheumatischer Erkrankung	Bei Erstdiagnose
	Im Verlauf
Rheumatische Erkrankung mit lymphozytärer Alveolitis, asymptomatisch und ohne manifeste ILD	Unklare Bedeutung
Manifeste ILD mit autoimmunen Markern (IPAF)	Unklare Bedeutung

### Diagnostik

Jeder Patient mit einer Erstdiagnose einer rheumatischen Erkrankung sollte pneumologisch vorgestellt und durch Lungenfunktion und HR-CT untersucht werden. Umgekehrt sollte jeder Patient mit einer ILD nach extrapulmonalen Symptomen gefragt werden, eine gründliche körperliche Untersuchung sowie eine Bestimmung relevanter Autoantikörper (Rheumafaktor, Anti-CCP-, ANA-, Anti-ds-DNA-, ENA-Antikörper) erhalten. Im Falle positiver Befunde ist immer eine rheumatologische Vorstellung angezeigt. Subklinische Alveolitiden bzw. ILD in der CT sollten im Verlauf kontrolliert werden [17].

Eine entsprechend vollständige Diagnostik erfolgt in der Praxis mutmaßlich nur unvollständig. Die Bedeutung einer gründlichen Fahndung nach extrapulmonalen Symptomen und wiederholter Autoantikörperbestimmungen bei initial negativen Befunden wurde kürzlich eindrucksvoll belegt [18].

Aus diesem Grund wird aktuell die Evaluation aller Patienten mit ILD in einer interdisziplinären Konferenz, mindestens bestehend aus Pneumologen, Radiologen, Pathologen und Rheumatologen empfohlen [9].

## Rheumatoide Arthritis (RA)

Die pulmonalen Manifestationen der rheumatoiden Arthritis umfassen am häufigsten das Lungenparenchym, die Atemwege sowie die Pleura [5]. Eine Vaskulitis und eine pulmonale Hypertonie sind demgegenüber nachgeordnet (Tab. 5). Die meisten Lungenmanifestationen treten innerhalb der ersten 5 Jahre nach Beginn der Erkrankung auf.

**Table 5:** 

Manifestation	Formen
Lungenparenchym	UIP
	NSIP
	OP
	AIP
	CEP
	Rheumaknoten
Pleura	Pleuritis mit und ohne Erguss
	Empyem
	Rheumaknoten, ggf. bronchopleurale Fisteln
	Pneumothorax
Atemwege	Schädigung des Vagus mit Stimmbandlähmung
	Dysfunktion der oberen Atemwege bei crycoarytenoider Arthritis
	Bronchiektasen
	Bronchiolitis obliterans
	Follikuläre Bronchiolitis
	Diffuse Panbronchiolitis
Lungenstrombahn	Pulmonale Hypertonie
	Thromboembolien

### Parenchymatöse Manifestationen

#### Epidemiologie

Eine ILD betrifft Männer häufiger als Frauen (im Verhältnis 2 bis 3:1) und tritt typischerweise bei RA im Erwachsenenalter in der fünften bis sechsten Lebensdekade auf („late onset“). Neben dem Lebensalter stellt das Rauchen einen zusätzlichen Risikofaktor dar. Seropositive Patienten (RF, besonders CCP) weisen ebenfalls häufiger eine ILD auf.

Die Häufigkeit hängt stark von der diagnostischen Methodik ab, wird aber auf ca. 50 % geschätzt. Etwa 30 % weisen in der HRCT eine subklinische ILD auf.

Die ILD tritt meist nach den Gelenkmanifestationen auf, kann aber auch gleichzeitig erscheinen oder dieser auch um Jahre vorausgehen. Als Faustregel kann gelten, dass beide Manifestationen in einem Zeitfenster von etwa 5 Jahren auftreten. Die Schweregrade von Gelenk- und Lungenerkrankung sind nur locker assoziiert.

#### Pathogenese

Die Pathogenese ist komplex und im Einzelnen ungeklärt. Es mehren sich jedoch die Zeichen, dass die RA in der Lunge beginnt. Verläufe mit positiven CCP-Antikörpern und ILD, jedoch ohne Gelenkmanifestationen sind dokumentiert. Zudem finden sich in den Lungen von Patienten mit RA reaktive lymphoide Gewebsformationen (BALT = „bronchus-associated lymphoid tissue“), die inflammatorische Zytokine und CCP-Antikörper produzieren. Rauchen fördert die Citrullinierung pulmonaler Proteine und somit die Induktion entsprechender Antikörper. Dies gilt insbesondere für einige HLA-Typen (B54, DQ1B*0601, B40, DR4). Die inflammatorischen Folgen eines Raucher-induzierten Epithelschadens potenzieren mutmaßlich die Autoantikörperinduktion und Fibrosierungen.

#### Diagnosestellung

Die Diagnosestellung erfolgt in erster Linie über die HR-CT. Differenzialdiagnostisch müssen Infektionen und toxische pulmonale Effekte der antirheumatischen Medikation ausgeschlossen werden. Eine bioptische Sicherung ist in der Regel nicht erforderlich.

**Figure 2:**
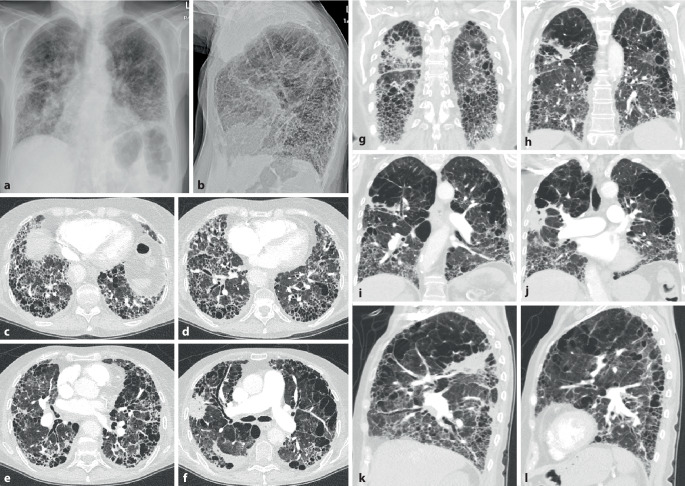

Die CT-Muster der ILD entsprechen mit ca. 40–60 % am häufigsten einer UIP [20, 21] (Abb. 2). Dies steht im Gegensatz zu anderen Autoimmunerkrankungen, bei denen die NSIP im Vordergrund steht. Diese wird hier lediglich in ca. 10–30 % der Fälle angetroffen (Abb. 5). Auch wenn die Prognose der Patienten mit RA und UIP-Muster besser ist als diejenige der Patienten mit IPF und UIP-Muster, bleibt sie doch schlechter als diejenige mit NSIP-Muster [19–21]. Dem entspricht eine geringere Anzahl an Fibroblastenherden im Lungengewebe [26].

**Figure 3:**
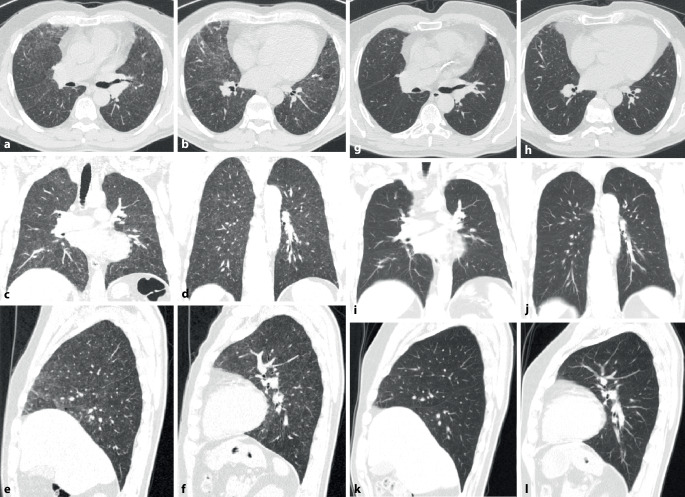

Konsolidierungen stellen häufig eine chronische organisierende Pneumonie COP (mit Bronchiolitis obliterans) dar. Disseminierte kleine zentrilobuläre Noduli als Ausdruck einer follikulären Bronchiolitis mit zentrilobulären Mattglasverschattungen in Kombination von Zonen gefangener Luft (air trapping) und einer Mosaikperfusion im Gefolge einer Bronchiolitis obliterans können ebenfalls Ausdruck einer pulmonalen Manifestation einer rheumatoiden Arthritis (Abb. 3) sein. Selten finden sich eine akute interstitielle Pneumonie (AIP) oder eine akute interstitielle Pneumonie (AIP) mit diffusem Alveolarschaden (DAD) und eine chronische eosinophile Pneumonie (CEP). In Ausnahmefällen werden auch eine lymphoide interstitielle Pneumonie (LIP) und eine desquamative interstitielle Pneumonie (DIP) gefunden.

Lungenfunktionell finden sich eine Restriktion sowie Reduktion des Transferfaktors (TCO) bei erhaltenem volumenkorrigiertem Transferfaktor (KCO). Eine Obstruktion oder gemischte Ventilationsstörung zeigt sich bei zusätzlicher Atemwegserkrankung. Die Reduktion der TCO ist ein unabhängiger prognostischer Prädiktor. Die geschilderten Befunde führen entsprechend ihres Ausmaßes zu einer unterschiedlich starken Beeinträchtigung der Funktionalität.

In der BALF findet sich bei Patienten mit RA häufig eine Lymphozytose mit Überwiegen der CD4-Zellen. Im Falle einer ILD kommt zusätzlich eine Neutrophile zur Erscheinung.

#### Therapie

Eine antiinflammatorische bzw. immunsuppressive Therapie ist bei Patienten mit eingeschränkter Funktionalität und/oder Symptomatik unabhängig vom HRCT-Muster indiziert, dies im Gegensatz zur IPF, in der eine immunsuppressive Therapie keine Wirkung erzielt.

Zur Therapie gibt es allerdings keine kontrollierten Studien. Steroide stehen traditionell im Vordergrund. Vorrangig werden sie heute bei NSIP- und OP-Mustern eingesetzt. Methotrexat scheint wirksam zu sein. Die dieser Substanz inhärente Lungentoxizität ist selten. Die Wirksamkeit von Cyclophosphamid und Azathioprin ist nicht zweifelsfrei belegt. Vielversprechende vorläufige Daten (Stabilisierung bei UIP, Besserung bei Non-UIP) gibt es zu Mycophenolat. Die Stellung von Therapien mit Anti-TNF‑α und Rituximab ist ungeklärt.

Die adjuvante Therapie entspricht derjenigen der nicht rheumatisch assoziierten ILD. Besonders wichtig erscheint dabei der Rauchverzicht.

Patienten mit progredienter ILD sollten für eine mögliche Transplantation evaluiert werden. Dies gilt insbesondere für Patienten mit UIP. Die Ergebnisse sind mit denen von Patienten ohne RA vergleichbar.

#### Prognose

Die ILD ist nach kardiovaskulären Ursachen die zweithäufigste Ursache des Todes von Patienten mit RA. Negative prognostische Faktoren sind Alter, männliches Geschlecht, UIP-Muster, Ausmaß der Fibrosierung und Reduktion der TCO. Die Prognose der RA-ILD-UIP ist besser als die IPF-UIP.

Die mittlere Überlebenszeit wurde mit 2,6 Jahre nach Diagnosestellung bei RA-ILD und 9,9 Jahre bei Patienten mit RA ohne ILD berichtet. Diese Zahlen sind jedoch abhängig von der Rate der RA-ILD mit UIP-Muster.

### Rheumaknoten

Rheumaknoten stellen eine Besonderheit der RA dar. Sie treten gehäuft bei Patienten mit länger bestehender RA sowie mit subkutanen Knötchen auf.

Histopathologisch zeichnen sich Rheumaknoten durch fibrinoide Verquellungen und zentrale Nekrosen des Bindegewebes aus, welche von palisadenartig angeordneten epitheloidzelligen mononukleären Bindegewebszellen umgeben sind und von einer Vaskulitis begleitet werden.

**Figure 4:**
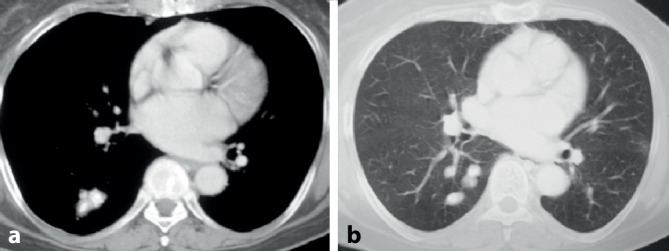

**Figure 5:**
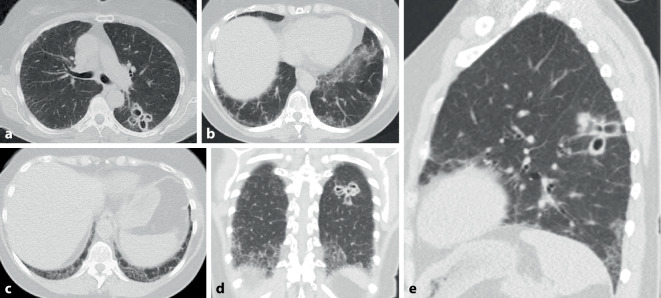

Rheumaknoten treten im Lungenparenchym oder in den interlobulären Septen bzw. subpleural einzeln oder multipel (Abb. 4 und 5) ohne (Abb. 4) oder mit zentralen Kavitationen (Abb. 5) auf. Ihre Größe variiert zwischen Millimetern und mehreren Zentimetern.

Rheumaknoten können sich spontan rückbilden, aber auch zunehmen, insbesondere unter Therapie. Dieses Paradoxon wird v. a. unter Methotrexat beobachtet.

Rheumaknoten als solche sind asymptomatisch. Charakteristisch ist jedoch ihre Neigung zur Einschmelzung (Abb. 5). Komplikativ können sich Hämoptysen oder ein Pleuraerguss, bronchopleurale Fisteln sowie ein Pneumothorax entwickeln.

Da das Rauchen gleichzeitig ein Risikofaktor für die Entwicklung von Rheumaknoten als auch von Lungenkrebs ist, müssen Rheumaknoten bei erstmaligem Auftreten von einem Tumor differenziert werden. Bei Herden bis 8 mm können dabei die Fleischner-Kriterien zur Anwendung kommen, ansonsten ist in der Regel eine histologische Sicherung erforderlich. In der PET-CT sind Rheumaknoten oft negativ, können in Abhängigkeit vom Status der Inflammation aber auch positiv ausfallen.

Eine Sonderform stellen Rheumaknoten bei Tätigkeit unter Tage mit Exposition gegen Kohle bzw. Silikate dar, ebenso bei Exposition auf Asbest (Caplan-Syndrom). Charakteristisch ist die plötzliche Entwicklung multipler Noduli. Diese können auch ohne Gelenkmanifestationen bzw. ILD auftreten.

Sie haben bis auf den Nachweis von Staubpartikeln histologisch eine identische Gestalt.

### Manifestationen der Atemwege

Diese sind insgesamt häufig und werden in obere und untere Atemwegsmanifestationen unterteilt.

Innerhalb der oberen Atemwege kann der N. laryngeus recurrens mit nachfolgender Stimmbandparese betroffen sein. Die Dysfunktion der oberen Atemwege bei crycoarytenoider Arthritis weist ein weites Spektrum an klinischen Symptomen auf. Selbst akute laryngeale Obstruktionen sind möglich.

Zu den Manifestationen der unteren Atemwege zählen v. a. Bronchiektasen und Bronchiolitiden.

Bronchiektasen treten ohne und im Rahmen der ILD (dann als Traktionsbronchiektasen) auf. Als solche sind sie mit bis zu 30 % häufig, jedoch selten symptomatisch. Sie können wie die ILD noch vor einer manifesten RA auftreten.

Bronchiolitiden sind mannigfach in ihrer Erscheinung und führen zu unterschiedlichen klinischen Verläufen. Die Bronchiolitis obliterans ist meist Raucher-assoziiert. Die follikuläre Bronchiolitis zeigt lymphoide Follikel in der Nähe von Bronchiolen. Beide manifestieren sich in der CT durch zentrilobuläre und/oder peribronchiale Noduli und eine Mattglasverschattung. Demgegenüber ist die diffuse Panbronchiolitis durch zusätzliche Tree-in-bud-Muster, Bronchiolo- bzw. Bronchiektasen charakterisiert.

### Pleurabeteiligung

Die Pleurabeteiligung ist eine der häufigsten Manifestationen (Abb. 3), insbesondere bei Männern mit lang dauernder rheumatischer Symptomatik und subkutanen bzw. pulmonalen Knoten („Rheumaknoten“).

Die Pleuritis manifestiert sich klinisch durch Pleuraschmerzen. Häufiger sind Pleuraergüsse.

Der Erguss stellt meist ein Exsudat mit hohen LDH-Werten >700–1000/UL dar. Charakteristisch sind neben einem pH < 7,3 (häufig um ca. 7,0) auch eine ausgeprägte Erniedrigung der Glukose <60 mg/dl (z. T. bis auf <30 mg/dl). Der niedrige pH korreliert dabei mit dem erhöhten Glukosemetabolismus bzw. konsekutiv erhöhtem Laktat und CO_2_-Konzentration. Die erniedrigte Glukosekonzentration ist wohl aber auch als Ausdruck eines gestörten Membrantransports. Zellulär dominieren neutrophile Granulozyten. Der RF ist häufig deutlich höher als der im Serum gemessene Wert.

Darüber hinaus sind auch sterile Empyeme nach Nekrose und Durchbruch eines Rheumaknotens, infizierte Empyeme mit bronchopleuraler Fistel und chylöse Ergüsse beschrieben.

Auf diesem Hintergrund ist die Differenzialdiagnose zu einem Empyem bzw. der Ausschluss einer gleichzeitigen Infektion von hoher Bedeutung.

Insbesondere kleinere Pleuraergüsse bei RA zeigen eine hohe spontane Rückbildungsrate. In einigen Fällen genügt die einmalige Thorakozentese mit kompletter Entleerung des Ergusses. Ansonsten ist aufgrund der Gefahr einer nicht mehr ausdehnungsfähigen Lunge in der Regel eine Steroidtherapie indiziert und erfolgreich.

### Lungenstrombahn

Etwa 20 % der Patienten mit RA weisen eine asymptomatische leichtgradige pulmonalarterielle Hypertonie auf. Die meisten Fälle mit pulmonaler Hypertonie treten jedoch in der Folge einer ausgedehnten ILD auf. Selten sind Hypertonien Folge einer Vaskulitis.

Die Therapie erfolgt entsprechend der Klasse-I-Hypertonie mit Vasodilatanzien (Endothelinantagonisten, Phosphodiesterase-V-Antagonisten).

Es besteht in Abhängigkeit von der Intensität der Inflammation (bzw. der extraartikulären Manifestationen) ein deutlich erhöhtes thromboembolisches Risiko.

## Systemischer Lupus erythematodes (SLE)

Der Lupus als vielgestaltigste rheumatische Erkrankung kann nahezu in jeder Form die Lunge befallen. Dennoch ergeben sich auch beim Lupus abgrenzbare typische Muster des Lungenbefalls [6]. Am häufigsten tritt ein Befall des Lungenparenchyms, der Pleura und der Gefäße [7] auf (Tab. 6).

**Table 6:** 

Manifestation	Formen
Lungenparenchym	Akute Pneumonitis
	NSIP
	OP
	LIP
	DAH
Pleura	Pleuritis mit und ohne Erguss
Lungenstrombahn	Thromboembolien
	Vaskulitis
	Pulmonale Hypertonie
Atemwege	Dysfunktion der oberen Atemwege bei crycoarytenoider Arthritis
	Bronchiektasen
	Bronchiolitis obliterans
Zwerchfell	Zwerchfellbefall

### Parenchymatöse Manifestationen

#### Akute Lupuspneumonitis

**Figure 6:**
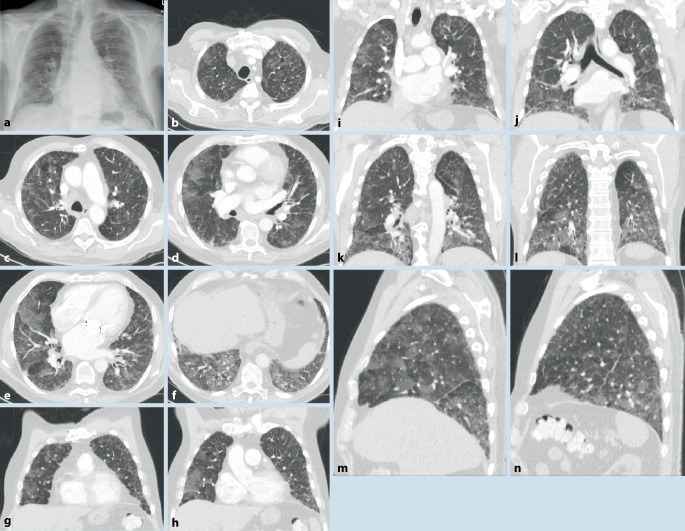

Die Lupuspneumonitis ist selten. Sie ist gekennzeichnet durch meist beidseitige diffuse Verschattungen oder Fleckschatten mit basaler Prädominanz (Abb. 6). Pleuraergüsse bestehen häufig zusätzlich. Klinisch umfasst die Symptomatik Fieber und eine ausgeprägte Oxygenierungsstörung. In schweren Fällen kommt es zu einem akuten Lungenversagen (ARDS).

Histopathologisch liegt der Lupuspneumonitis ein diffuser Alveolarschaden (DAD) mit interstitiellem Ödem und Bildung hyaliner Membranen zugrunde.

Die Diagnose gestaltet sich aufgrund der schweren Oxygenierungsstörung mit eingeschränkten Optionen der Histologiegewinnung meist sehr schwierig. Die Differenzialdiagnose ist breit und umfasst eine kardiale Dekompensation, eine interstitielle Einlagerung bei Nierenversagen, infektiöse Pneumonien sowie die DAH.

Die Therapie besteht in der Gabe hoch dosierter Steroide (1 mg/kg KH) mit frühzeitiger Einführung eines „Steroidsparers“. In schweren oder therapieresistenten Fällen ist die Gabe von Hochdosissteroiden plus Cyclophosphamid ggf. in Kombination mit Plasmapheresen indiziert. Neuere Therapieansätze bestehen in Rituximab und Belimumab.

#### Chronische ILD

**Figure 7:**
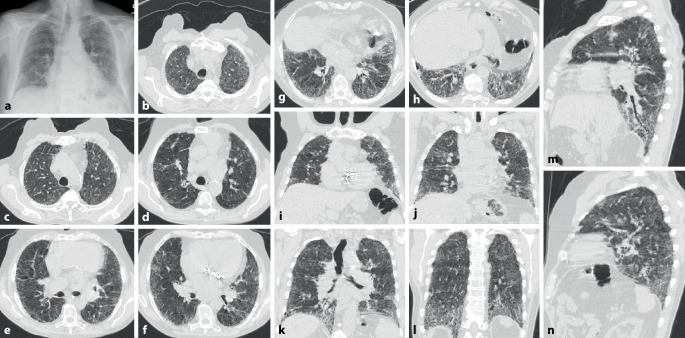

Eine chronische ILD kann beim systemischen Lupus erythematodes aus einer akuten hervorgehen (Abb. 7) oder primär chronisch entstehen.

Die CT-Muster unterscheiden sich je nachdem, ob der Patient symptomatisch ist oder nicht.

Bei symptomatischen Patienten überwiegen Mattglasverschattungen und Pleuraverdickungen, zudem können interlobuläre Septenverdickungen oder konsolidierende Fleckschatten vorkommen. Sind die Patienten asymptomatisch, stehen die Retikulationen sowie der Befall der Atemwege im Vordergrund.

Grundsätzlich werden Muster der NSIP und LIP sowie auch Honigwaben (Abb. 7) gesehen.

Lungenfunktionell sind eine Restriktion sowie Reduktion der Diffusionskapazität der häufigste Befund. In der BALF überwiegt eine Lymphozytose.

#### Diffuse alveoläre Hämorrhagie (DAH)

**Figure 8:**
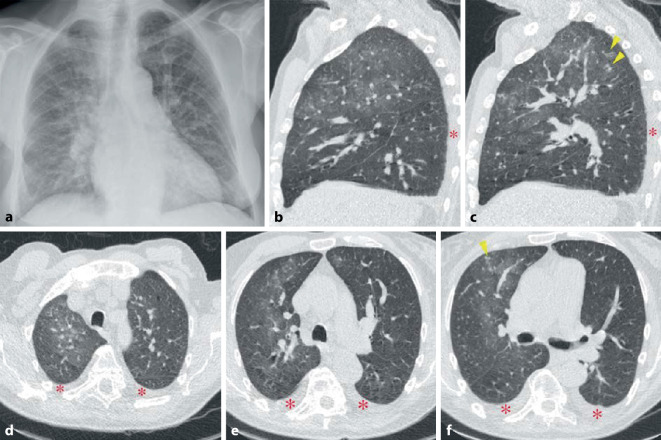

Eine DAH (Abb. 8) ist selten und entsteht meist akut. Sie kommt auch als einzige pulmonale Manifestation eines SLE vor. Die Hämorrhagien resultieren aus einer Vaskulitis. Hämoptysen/Hämoptoe bestehen nur in ca. der Hälfte der Fälle. In der CT zeigen sich typische Füllungsmuster. Die Diagnose kann bronchoskopisch erfolgen. Typisch sind die Blutreste bzw. nachlaufende Blutungen auf den großen Atemwegen bzw. bei makroskopisch nicht erkennbaren Blutungen die mit jeder Lavagefraktion zunehmende blutig tingierte BALF. Mikroskopisch zeigen sich massenhaft Siderophagen.

### Pleurabeteiligung

Pleuraergüsse kommen in 30 % der Patienten vor. Selten (maximal 5 %) sind diese Erstmanifestation eines SLE, dann jedoch meist in Form einer Polyserositis. Der Pleuraerguss besteht meist beidseitig, nicht selten im Rahmen einer Polyserositis. Im Unterschied zur RA findet sich zwar ebenfalls ein Exsudat, aber mit höheren Glukosewerten (>60 mg/dl) und niedrigeren LDH-Werten (>500 U/L). ANA- und ds-DNA-Antikörper können gefunden werden. Die Pleuraergüsse sprechen in der Regel sehr gut auf nichtsteroidale Antiphlogistika als auch Steroide an. Sie stellen keine prognostische Einschränkung dar.

### Lungenstrombahn

Mit Thromboembolien muss in ca. 25 % der Patienten mit Lupus gerechnet werden. Im Rahmen des Antiphospholipidsyndroms sind auch Mikrothrombosen sowie arterielle Thrombosen möglich.

Die pulmonale Hypertonie (Abb. 7) kann eine Folge stattgehabter, nicht (vollständig) lysierter Thromben oder auch einer Vaskulitis der kleinen Gefäße sein (dies v. a. auch im Rahmen des Antiphospholipidsyndroms). Ein weiterer Risikofaktor ist der Nachweis eines Antitopomerase-1-Antikörpers. Bei nicht fixierter pulmonaler Hypertonie ist eine immunsuppressive Therapie wirksam.

Nach Ausschluss einer chronisch thromboembolischen pulmonalen Hypertonie (CTEPH) handelt es sich um eine Klasse-I-Hypertonie, die mit Vasodilatanzien behandelt werden kann (Endothelinantagonisten, Phosphodiesterase-V-Antagonisten).

Eine Besonderheit stellt das akute reversible Hypoxämiesyndrom dar. Bei diesem kommt es zu einer schweren Oxygenierungsstörung ohne Lungenparenchymbefall. Einzig eine Störung der Diffusionskapazität ist zusätzlich auffällig. Es handelt sich um eine Aggregation von Komplement-aktivierten Neutrophilen im Lungenstrombett. Steroide sind wirksam.

### Andere Befallsmuster

Der Atemmuskel- bzw. Zwerchfellbefall manifestiert sich über einen Zwerchfellhochstand beidseits mit begleitenden basalen Dystelektasen und führt zum Syndrom der „schrumpfenden“ oder „verschwindenden“ Lunge. Ursächlich besteht eine muskuläre Zwerchfelllähmung. Im Gegensatz zur PM/DM/Jo‑1 besteht kein Lungenparenchymbefall. Auch hier ist eine immunsuppressive Therapie angezeigt. Steroide sind Mittel der Wahl.

Der Befall der Atemwege ist selten.

## Progressive systemische Sklerose (Sklerodermie, PSS)

Die Mehrzahl der Patienten mit PSS weisen pulmonale Manifestationen auf, auch wenn diese nur Rang 4 der Häufigkeit des Organbefalls hinter der Haut, den kleinen peripheren Gefäßen und dem Ösophagus belegen. Die Varietät der Lungenmanifestationen ist geringer als bei RA und SLE (Tab. 7). Der Pulmonalbefall ist prognostisch vergleichbar ernst wie der Nierenbefall.

**Table 7:** 

Manifestation	Formen
Lungenparenchym	NSIP
	UIP
	DAD
Lungenstrombahn	Pulmonale Hypertonie^a^
	DAH
Pleura	Pleuritis mit und ohne Erguss

^a^Insbesondere bei CREST-Syndrom

### Lungenparenchymbefall

#### Präsentation und Diagnostik

Etwa 50–85 % der Patienten mit PSS weisen zum Zeitpunkt der Erstdiagnose eine ILD auf. Ein Lungenparenchymbefall kann ähnlich der RA der Diagnose der Sklerodermie zeitlich deutlich vorweggehen. Umgekehrt ist bei normalem Initialbefund die Entwicklung einer ILD mit 15 % relativ selten. Ein Scl-70-Antikörper, der in der Mehrzahl der Patienten mit PSS vorliegt, ist mit einer ILD assoziiert.

**Figure 9:**
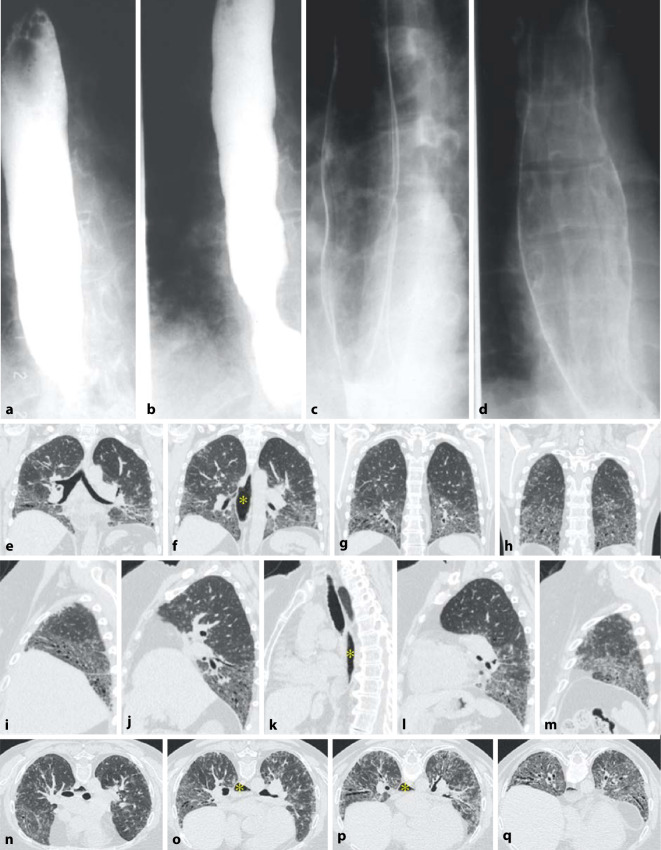

**Figure 10:**
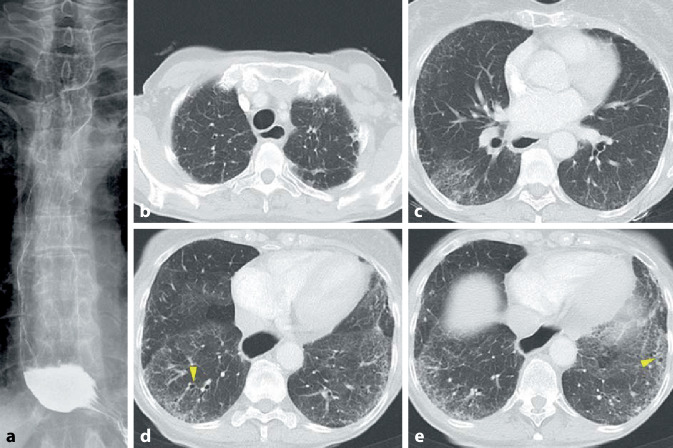

In der CT können ein NSIP- (Abb. 9) oder (seltener) ein UIP-Muster bzw. probable UIP-Muster (Abb. 10) vorliegen. Zusätzlich können Pleuraverdickungen sowie intrathorakale Lymphknotenvergrößerungen imponieren.

Darüber hinaus werden selten eine OP und eine DAH (mit Vaskulitis bzw. Kapillaritis) gesehen.

Eine Besonderheit stellen beim CREST-Syndrom kalzifizierte Granulome dar.

Das Lungenfunktionsmuster entspricht überwiegend einer restriktiven Ventilationsstörung mit unterschiedlich ausgeprägter Gasaustauschstörung.

In der BALF finden sich je nach Aktivität eine Lymphozytose, Neutrophilie, aber auch Eosinophilie. Eine Neutrophilie ist prognostisch nachteilig und korreliert mit dem Verlust an Vitalkapazität (FVC) und Diffusionskapazität.

#### Therapie

Die PSS ist die einzige rheumatische Erkrankung mit ILD, für die gesicherte Therapien zur Verfügung stehen. Cyclophosphamid verbessert Parameter der Lungenfunktion (FVC), die Dyspnoe, das Skleroderm sowie die Lebensqualität. Die Effekte halten 2 Jahre an, verbrauchen sich jedoch nach dieser Zeit (bis auf die Effekte auf die Dyspnoe) [22, 23]. Mycophenolat weist eine vergleichbare Wirkung auf, ist jedoch besser verträglich [24].

OP und DAH werden durch Steroide (ggf. plus Steroidsparer) behandelt.

Vom Lungenparenchymbefall muss die rezidivierende Aspirationspneumonie im Rahmen der Ösophagusmotilitätsstörung abgegrenzt werden. Es ist zurzeit noch unklar, ob diese Aspirationsereignisse im Zusammenhang mit der Entwicklung einer ILD stehen.

### Lungenstrombahn

Eine pulmonale Hypertonie tritt in ca. 30 % der Fälle mit CREST-Syndrom auf und ist häufiger als bei der diffusen Sklerodermie (ca. 10 %). Bei Patienten mit Antikörpern gegen Zentromere und Histone im Serum liegt ein erhöhtes Risiko für die Entwicklung einer pulmonalen Hypertonie vor. Bei schwerem Raynaud-Syndrom besteht fast immer eine pulmonale Hypertonie.

Sie kann ohne und mit ILD auftreten. Histologisch zeigen sich bei der alleinigen pulmonalarteriellen Hypertonie Gefäßobliterationen, bei derjenigen im Rahmen einer ILD kommen eine Intima- und Mediaproliferation zur Darstellung. Nekrosen im Rahmen einer Vaskulitis werden nicht angetroffen.

Die Therapie erfolgt entsprechend der Klasse-I-Hypertonie mit Vasodilatanzien (Endothelinantagonisten, Phosphodiesterase-V-Antagonisten).

### ILD und systemische Sklerose sine Skleroderma

Diese seltene, aber wichtige Gruppe ist zu vermuten bei Vorliegen folgender Kriterien: ANA, Teleangiektasie, Raynaud-Syndrom mit pathologischer Kapillaroskopie, gastroösophagealer Reflux und Perikarderkrankung [25].

## Polymyositis/Dermatomyositis/Jo-1- bzw. Antisynthetasesyndrom (ASS)

Bei dieser Erkrankung stehen neben der ILD besonders die Komplikationen im Vordergrund: Aspirationspneumonien aufgrund des pharyngealen und ösophagealen Befalls, infektiöse Pneumonien aufgrund des Zwerchfellbefalls sowie sekundäre Tumorerkrankungen stehen dabei im Vordergrund (Tab. 8).

**Table 8:** 

Manifestation	Formen
Lungenparenchym	NSIP
	DAD
	OP
Zwerchfell	Zwerchfellbefall

### Lungenparenchymbefall

Dieser wird v. a. bei Dermatomyositis beobachtet.

Im Rahmen der ILD wird hauptsächlich ein NSIP- und UIP-Muster beobachtet. Zudem ist v. a. auch die organisierende Pneumonie (OP) relevant. Im Falle von Konsolidierungen liegt häufig jedoch ein diffuser Alveolarschaden (DAD) vor.

Differenzialdiagnostisch bedeutsam sind wie bei PSS Aspirationspneumonien, dazu aber auch infektiöse Pneumonien in der Folge der Hypostase bei Zwerchfellhochstand.

Bronchoskopisch finden sich eine Lymphozytose als auch eine Neutrophilie.

### Befall der Atemmuskel- bzw. Zwerchfellmuskulatur

Ein symptomatischer Befall wird bei ca. 10 % der Patienten gesehen. Ein (noch) asymptomatischer Befall ist mutmaßlich viel höher als früher angenommen.

Die klinische Präsentation entspricht derjenigen im Rahmen des SLE.

Infektiöse Pneumonien im Rahmen der basalen Hypostase sind eine häufige Komplikation.

### Jo-1-Antisynthetasesyndrom (ASS)

**Figure 11:**
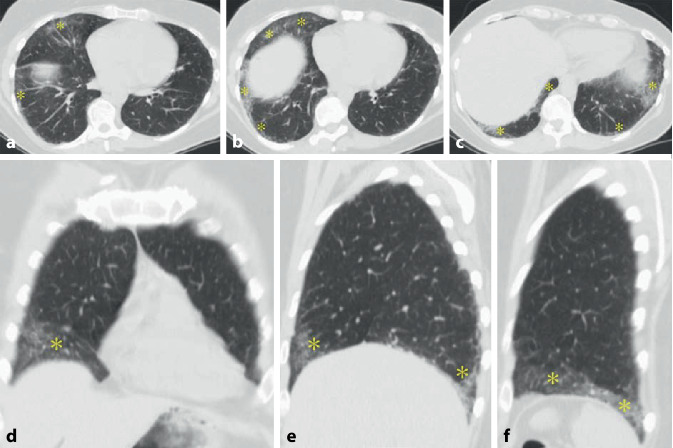

Die ILD kommt in ca. 60 % der Patienten vor (Abb. 11). Sie scheint wenig progredient bzw. zumindest unter Therapie stationär zu verlaufen [27]. Eine ILD wird auch beim Antisynthetasesyndrom (ASS) ohne Jo-1-Antikörper beobachtet.

**Figure 12:**
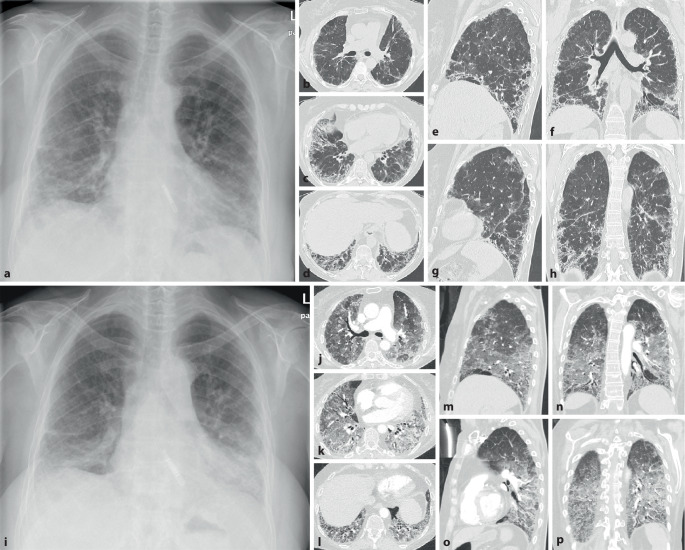

Als Antisynthetasesyndrom wird eine überwiegend in Assoziation mit einer Dermatomyositis auftretende Kombination aus Myositis, interstitieller Pneumonitis mit fibrosierender Alveolitis, nichterosiver Arthritis mit Synovitis und Raynaud-Syndrom [28] sowie der häufigen Assoziation mit einem Sicca-Syndrom, Fieber und einem Karpaltunnelsyndrom bezeichnet. Bei Patienten mit Myositis ist die Lunge häufig beteiligt, und das Vorhandensein von Anti-Aminoacyl-t-RNA-Synthetase(Anti-ARS)-Antikörpern markiert das Vorhandensein oder sagt die Entwicklung einer interstitiellen Lungenerkrankung voraus. Ein ausgeprägtes klinisches Antisynthetasesyndrom ist durch das Vorhandensein von Anti-ARS-Antikörpern, Myositis, interstitieller Lungenerkrankung, Fieber, Arthritis, Raynaud-Phänomen und sog. „mechanischen Händen“ gekennzeichnet. Der häufigste Anti-ARS-Antikörper ist Anti-Jo‑1. Kürzlich beschriebene Anti-ARS-Antikörper könnten einen Phänotyp bezeichnen, der sich von dem von Anti-Jo-1-positiven Patienten unterscheidet und durch eine geringere Inzidenz von Myositis und eine höhere Inzidenz von interstitiellen Lungenerkrankungen gekennzeichnet ist [28]. Bei Patienten mit interstitiellen Lungenerkrankungen im Zusammenhang mit dem Antisynthetasesyndrom ist die Reaktion auf immunsuppressive Medikamente im Allgemeinen, aber nicht überall, günstig. Bei vielen Patienten mit einer Antisynthetasesyndrom-bedingten interstitiellen Lungenerkrankung beginnt die Dyspnoe allmählich über Monate. Bei einer Untergruppe von Patienten stellt sich der Beginn von interstitieller Lungenerkrankung, Fieber und respiratorischer Insuffizienz jedoch abrupt innerhalb weniger Tage oder Wochen dar (Abb. 12).

## Pulmonale Manifestationen des Sjögren-Syndroms

Die Exokrinopathie, die sich primär auf Tränen- und Speicheldrüsen bezieht, kann in ca. 10 % der Fälle zu einer Beteiligung der Lunge führen (Tab. 9).

**Table 9:** 

Manifestation	Formen
Atemwege	Trockene Atemwege
	Rezidivierende Atemwegsinfektionen
	Bronchiektasen
Lungenparenchym	Lymphozytäre Alveolitis
	Organisierende Pneumonie
	Pseudolymphom
	Lymphom
Lungenstrombahn	Pulmonale Hypertonie (selten)

In ca. 90 % der Fälle besteht ein positiver Rheumafaktor.

**Figure 13:**
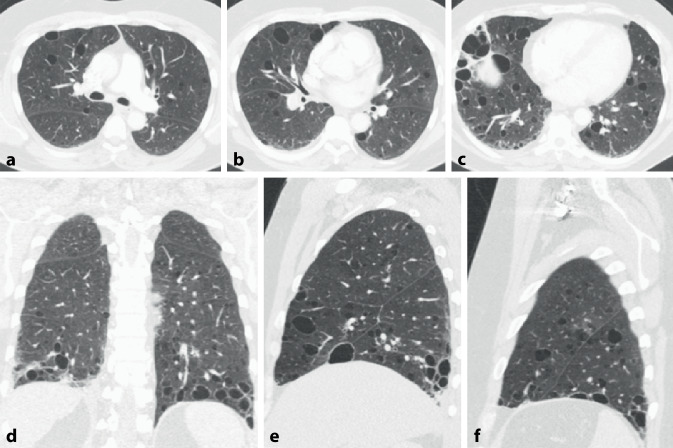

Die Lunge kann in mehrfacher Form betroffen sein:

- lymphozytäre Alveolitis bzw. lymphoide interstitielle Pneumonitis „LIP“ (Abb. 13),
- trockene Atemwege mit nachfolgendem erhöhtem Risiko für Pneumonien, langfristig auch Bronchiektasien,
- organisierende Pneumonie,
- Pseudolymphom oder auch malignes Lymphom.

Ein Vorkommen von NSIP und UIP ist beschrieben, bleibt jedoch die Ausnahme.

Die lymphozytäre Alveolitis kann asymptomatisch sein und auch mit einer unauffälligen CT einhergehen oder durch ein mikronoduläres CT-Muster mit und ohne Milchglasverschattungen, welche häufig mit einer Neutrophilie in der BAL assoziiert sind, einhergehen. Im weiteren Verlauf können sich in der CT Lungenzysten (Abb. 13) nachweisen lassen. Verdickungen der Atemwege mit Ausbildung von kleineren Bronchiektasien sind häufig.

Bioptisch zeigt sich häufig eine Lymphozyteninfiltration in der Bronchialwand und im Interstitium. Pseudolymphome und maligne Lymphome, welche sich CT-morphologisch als Fleckschatten oder Konsolidierungen manifestieren können, unterscheiden sich durch die klonale Präsenz unreifer Lymphozyten und ggf. den Befall der regionalen Lymphknoten.

Eine wirksame Therapie ist nicht gesichert. Je nach Schweregrad kann ein Versuch mit Steroiden und anderen Immunsuppressiva erfolgen.

## Pulmonale Manifestationen der „mixed connective tissue disease“ (MCTD)

Die Häufigkeit einer ILD bei MCTD ist unbekannt. Es zeigen sich häufig interlobuläre Septenverdickungen sowie Noduli, aber auch Honigwabenbildungen. Seltener (ca. 10 %) werden Milchglasverschattungen angetroffen. Die CT-Muster sind nicht einheitlich, dürften jedoch überwiegend der NSIP und UIP entsprechen.

Entsprechend Ergebnissen der Lungenfunktion sind häufiger die kleinen Atemwege befallen. Bisher konnte jedoch kein bildgebendes Korrelat dafür identifiziert werden.

In ca. 80 % der Fälle besteht ein Ösophagusbefall mit nachfolgender Dysmotilität. Daher sind chronische Aspirationspneumonien häufig.

Pleuritiden bzw. Pleuraergüsse sind mit ca. 30 % häufig. Pleuraergüsse können auch Primärmanifestation der MCTD sein. In der CT zeigen sich in ca. 60–70 % Pleuraverdickungen. Pleuritis und Pleuraergüsse sprechen gut auf Steroide an.

Die Ausbildung einer pulmonalen Hypertonie kann vielfache Ursachen haben. Sie kann sich sekundär bei bestehender ILD ausbilden oder im Rahmen einer plexigenen Angiopathie (Intimaproliferation der Arteriolen). Die Therapie erfolgt entsprechend der Klasse-I-Hypertonie mit Vasodilatanzien (Endothelinantagonisten, Phosphodiesterase-V-Antagonisten).

Die pulmonalen Manifestationen finden sich in Tab. 10 zusammengefasst.

**Table 10:** 

Manifestation	Formen
Atemwege	Befall der kleinen Atemwege
Lungenparenchym	NSIP
	UIP
	Chronische Aspirationspneumonie
Pleura	Pleuritis, Pleuraerguss
Lungenstrombahn	Pulmonale Hypertonie

## Pulmonale Manifestationen der axialen Spondyloarthropathien und insbesondere der ankylosierenden Spondylitis (Morbus Strümpell-Marie-Bechterew)

Bei der ankylosierenden Spondylitis sind Lungenparenchymbefall und eine Ankylosierung der kleinen Wirbel- und Rippengelenke führend (Tab. 11).

**Table 11:** 

Manifestation	Formen
Lungenparenchym	Zystisch-fibrotischer Umbau der Oberlappen
Thoraxskelett	Ankylosierung der kleinen Wirbel und Rippengelenke mit Thoraxstarre und Thoraxverkleinerung

### Lungenparenchymbefall

**Figure 14:**
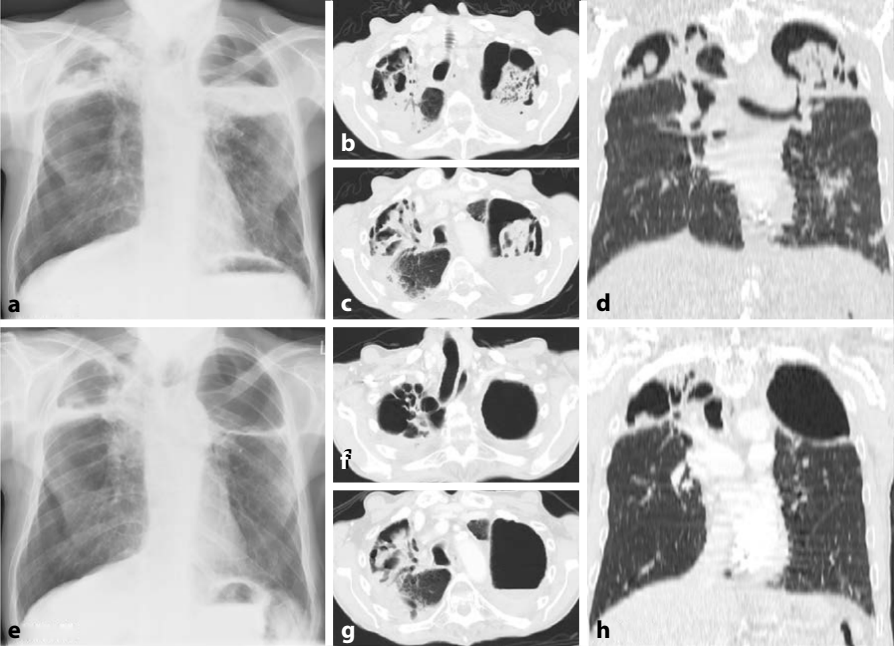

Als einzige rheumatische Erkrankung weist die ankylosierende Spondylitis ein besonderes und typisches Befallsmuster der Lunge auf. Dabei handelt es sich um ein Befallsmuster, welches die Oberlappen betrifft und durch einen zystisch-fibrotischen Umbau gekennzeichnet ist (Abb. 14). Es handelt sich jedoch um eine seltene Manifestation (ca. 1 %). Sie dürfte durch frühzeitige effektive Therapie der Grunderkrankung heute noch deutlich seltener sein.

Differenzialdiagnostisch ist immer eine Tuberkulose abzugrenzen.

Eine typische Komplikation betrifft dann im Verlauf die Ausbildung von Aspergillomen (Abb. 14) oder auch einer chronisch nekrotisierenden Aspergillose (CNA).

Aspergillome sind nur im Falle von Lungenblutungen therapiepflichtig und dann durch interventionelle DSA mit Coiling (Resektion möglichst vermeiden) und durch eine Aspergillus-spezifische Suppressionstherapie anzugehen. Die CNA ist eine semiinvasive Verlaufsform der Aspergillose und muss in jedem Fall mit Antimykotika (Azolen, Amphotericin B) behandelt werden.

### Ankylosierung der kleinen Wirbel- und Rippengelenke

Über diesen Oberlappenbefall hinaus kommt es bei andauernder unkontrollierter ankylosierender Spondylitis zu einer Thoraxstarre und Thoraxverkleinerung. Diese bewirkt eine (meist mäßiggradige) restriktive Ventilationsstörung. Dennoch besteht, ganz im Gegensatz zur Kyphoskoliose, meist keine relevante funktionelle Einschränkung. Dies liegt darin begründet, dass die Thoraxstarre die Lunge meist bei normalen Lungenvolumina erfasst, sodass die dynamischen Lungenvolumina unbeeinträchtigt bleiben; im Gegensatz dazu kommt es im Rahmen der Kyphoskoliose zu einer erheblichen Minderbelüftung und Reduktion der Lungenvolumina, die im Verlauf nur unzureichend durch verstärkte Zwerchfellarbeit kompensiert werden können.

Bei gleichzeitig bestehender COPD mit Lungenemphysem kommt es allerdings zu einer Konstellation einer Thoraxstarre, die auf eine erhebliche, raumfordernde Überblähung trifft. Im Ergebnis entsteht eine besonders ungünstige Atemmechanik. In der Folge kann es daher zu einem chronischen Ventilationsversagen kommen. In diesen Fällen ist eine nichtinvasive Heimbeatmung (NIV) angezeigt.

### Pulmonale Manifestationen bei enteropathischen Spondyloarthritiden

**Figure 15:**
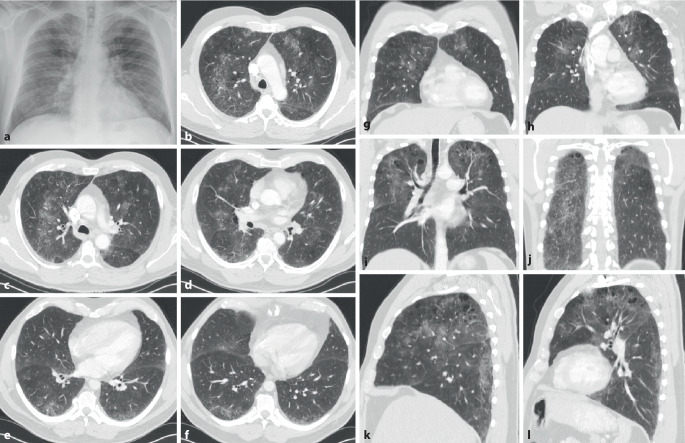

Auch die enteropathischen Spondyloarthritiden im Rahmen einer Colitis ulcerosa und eines Morbus Crohn (Abb. 15) sind mit einer Vielzahl von systemischen Manifestationen auch an der Lunge assoziiert [29, 30]. Lunge und Magen-Darm-System stammen aus dem primitiven Darm, und sie haben die gleichen pathogenetischen Veränderungen bei diesen Patienten. Die wichtigsten Muster der Lungenerkrankung, die mit einer entzündlichen Darmerkrankung assoziiert sind, erstrecken sich auf Pleuritiden, Atemwegserkrankungen, interstitielle Lungenerkrankungen, nekrobiotische Knoten, pulmonale Eosinophilie, thromboembolische Erkrankungen, Vaskulitis, granulomatöse Lungenerkrankungen. Kolektomien können die Symptome der Atemwege verschlimmern, während Therapien mit Sulfasalazinen, Mesalaminen, Methotrexat und Anti-TNF‑α sich auch günstig auf die Lungenveränderungen auswirken. Latente Lungenfunktionsstörungen treten entweder bei Lungenfunktionstests in Erscheinung oder lassen sich durch eine bronchoalveoläre Lavage aufdecken, auch wenn kein klinischer Nachweis einer Atemwegserkrankung vorliegt.

## Rezidivierende Polychondritis

Bei der rezidivierenden Polychondritis besteht eine chronische, episodisch aktivierte, systemische Inflammation mit ausgedehntem Befall der kartilaginären Strukturen des äußeren Ohrs, der Gelenke, der Nase und der Atemwege.

Eine lokale oder systemische Vaskulitis mit Befall der mittleren und großen Gefäße ist häufig. Entsprechend bilden ca. 25 % kardiovaskuläre Komplikationen in Form von Klappenvitien, Aneurysmata oder Karditiden aus.

In etwa 25 % der Fälle besteht gleichzeitig eine RA, ein SS, ein SLE oder eine AS.

Der Befall der oberen Atemwege führt zu einer Destruktion der laryngotrachealen Strukturen mit der Folge schwerwiegender Atemwegsstenosen. Therapeutisch steht neben einer intensiven Immunsuppression eine interventionelle Therapie zur Freihaltung der Atemwege im Vordergrund. Bei trachealem oder tracheobronchialem Befall ist häufig die Implantation eines Stents indiziert.

## Andere rheumatische Erkrankungen

Lungenmanifestationen sind kasuistisch oder in kleinen Fallserien auch für die Psoriasisarthritis und den Morbus Behçet beschrieben. Aufgrund der unklaren Datenlage müssen mögliche Fälle aktuell individuell definiert und behandelt werden.

## Lungentoxizität von Substanzen in der Therapie der rheumatischen Erkrankungen

Viele in der Therapie rheumatischer Erkrankungen eingesetzte Substanzen haben eine potenzielle Lungentoxizität. Diese muss in der Differenzialdiagnose zur ILD in jedem Fall Berücksichtigung finden.

Eine kurze Übersicht über mögliche bzw. typische Schädigungsmuster wichtiger Substanzen wird unten aufgeführt.

In Ergänzung dazu sei auf die Internetseite www.pneumotox.com hingewiesen, die eine systematische Sammlung aller in der Weltliteratur publizierten Manifestationen von Toxizitäten entsprechender Substanzen (grundsätzlich auch aller Medikamente) bietet.

### Methotrexat

Methotrexat ist die wichtigste Erstliniensubstanz in der Therapie der RA.

Eine akute bzw. subakute Hypersensitivitätspneumonitis tritt in ca. 1–7 % der Fälle auf. Typischerweise wird sie meist innerhalb des ersten Behandlungsjahres gesehen.

In der CT zeigen sich diffuse Fleckschatten bzw. Konsolidierungen. In transbronchialen Biopsien werden nicht nekrotisierende Granulome und eine Eosinophilie beobachtet.

Häufig führt allein das Absetzen der Medikation zu einer kompletten Remission, bei ausgedehnten Befunden können aber auch Steroide zum Einsatz kommen.

Neben dieser akuten ist auch eine chronische Verlaufsform beschrieben, es scheint sich dabei jedoch nach neueren Daten eher um eine ILD im Rahmen der Erkrankung selbst zu handeln.

Regelmäßige Lungenfunktionsprüfungen sind zur Früherkennung einer Methotrexat-Toxizität nicht geeignet und werden entsprechend nicht empfohlen.

### Leflunomid

Für Leflunomid ist die Entwicklung einer ILD bzw. einer akuten Exazerbation einer ILD beschrieben. Pathogenetisch liegt wahrscheinlich die Produktion eines aktiven Metaboliten zugrunde, der die Transition von pulmonalen Epithelzellen zu Myofibroblasten induzieren kann. Dies scheint jedoch nur im Rahmen einer genetischen oder toxischen Prädisposition möglich.

Asiatische Populationen sind entsprechend vulnerabler als europäische (Leflunomid-assoziierte ILD 1 % vs. <0,1 %).

### TNF-α-Inhibitoren

Aktuell ist unklar, ob TNF-α-Inhibitoren das Potenzial haben, eine ILD zu induzieren bzw. zu einer Exazerbation einer bestehenden ILD zu führen. Ein erschwerender Faktor in der Wertung vorliegender Daten besteht darin, dass die meisten Patienten bereits eine vorbestehende Medikation erhalten haben bzw. ein relevanter Teil schon eine ILD aufweist.

Das Risiko einer Tuberkulose und nichttuberkulöser Mykobakteriosen ist deutlich erhöht, sofern keine präventive Therapie vor Einleitung der Behandlung erfolgt ist.

### Andere Substanzen

Rituximab scheint bei Patienten mit rheumatologischen Erkrankungen keine lungentoxischen Wirkungen zu zeitigen.

Gold ist nur selten mit einer Lungentoxizität assoziiert (maximal 1 %).

Sulfasalazin kann eosinophile Pneumonien induzieren, die nach Absetzen der Substanz spontan remittieren.

## Fazit für die Praxis

- Lungenbeteiligungen bei rheumatologischen Erkrankungen sind häufig und können jede anatomische Struktur betreffen (Trachea, Bronchien, Bronchiolen, Alveolen, Interstitium, Pleura, Zwerchfellmuskulatur).
- Lungenbeteiligungen sind prognostisch von hoher Relevanz und begründen Therapieentscheidungen.
- Daher sollte bei rheumatologischen Erkrankungen eine Evaluation eines möglichen Lungenbefalls bei Erstdiagnose und im Verlauf erfolgen.
- Die Kenntnis der HR-CT-Muster ist für die adäquate Klassifikation des interstitiellen Lungenbefalls unabdingbar.
